# Concept development of a mainstream deammonification and comparison with conventional process in terms of energy, performance and economical construction perspectives

**DOI:** 10.3389/fmicb.2023.1155235

**Published:** 2023-04-11

**Authors:** Dheeraja Cheenakula, Kai Griebel, David Montag, Markus Grömping

**Affiliations:** ^1^Institute NOWUM-Energy, FH Aachen, University of Applied Sciences, Jülich, Germany; ^2^Institute of Environmental Engineering, RWTH Aachen, Aachen, Germany

**Keywords:** anammox, energy efficiency, mainstream deammonification, nitrogen elimination, wastewater

## Abstract

Deammonification for nitrogen removal in municipal wastewater in temperate and cold climate zones is currently limited to the side stream of municipal wastewater treatment plants (MWWTP). This study developed a conceptual model of a mainstream deammonification plant, designed for 30,000 P.E., considering possible solutions corresponding to the challenging mainstream conditions in Germany. In addition, the energy-saving potential, nitrogen elimination performance and construction-related costs of mainstream deammonification were compared to a conventional plant model, having a single-stage activated sludge process with upstream denitrification. The results revealed that an additional treatment step by combining chemical precipitation and ultra-fine screening is advantageous prior the mainstream deammonification. Hereby chemical oxygen demand (COD) can be reduced by 80% so that the COD:N ratio can be reduced from 12 to 2.5. Laboratory experiments testing mainstream conditions of temperature (8–20°C), pH (6–9) and COD:N ratio (1–6) showed an achievable volumetric nitrogen removal rate (VNRR) of at least 50 gN/(m^3^∙d) for various deammonifying sludges from side stream deammonification systems in the state of North Rhine-Westphalia, Germany, where m^3^ denotes reactor volume. Assuming a retained N_organic_ content of 0.0035 kgN_org._/(P.E.∙d) from the daily loads of N at carbon removal stage and a VNRR of 50 gN/(m^3^∙d) under mainstream conditions, a resident-specific reactor volume of 0.115 m^3^/(P.E.) is required for mainstream deammonification. This is in the same order of magnitude as the conventional activated sludge process, i.e., 0.173 m^3^/(P.E.) for an MWWTP of size class of 4. The conventional plant model yielded a total specific electricity demand of 35 kWh/(P.E.∙a) for the operation of the whole MWWTP and an energy recovery potential of 15.8 kWh/(P.E.∙a) through anaerobic digestion. In contrast, the developed mainstream deammonification model plant would require only a 21.5 kWh/(P.E.∙a) energy demand and result in 24 kWh/(P.E.∙a) energy recovery potential, enabling the mainstream deammonification model plant to be self-sufficient. The retrofitting costs for the implementation of mainstream deammonification in existing conventional MWWTPs are nearly negligible as the existing units like activated sludge reactors, aerators and monitoring technology are reusable. However, the mainstream deammonification must meet the performance requirement of VNRR of about 50 gN/(m^3^∙d) in this case.

## 1. Introduction

Deammonification is an emerging technology for biological nitrogen (N) removal. It offers advantages for the energy and resource efficiency of municipal wastewater treatment plants (MWWTP) e.g., carbon (C). The biological process of deammonification consists of two steps. It is based on the partial nitritation of ammonium (NH_4_^+^) to nitrite (NO_2_^−^) and the subsequent anaerobic ammonium oxidation (anammox) combined with NO_2_-consumption to elemental N (Mao et al., 2017). While the aerobic, nitrite-oxidizing bacteria require oxygen for the partial conversion of ammonium, the anammox reaction takes place under anoxic conditions. This has the advantage of 60% lower oxygen demand and thus a high potential for energy savings compared to the conventional activated sludge process using nitrification/−denitrification (Lucy Pugh, 2017). Both reactions in deammonification are carried out by autotrophic microorganisms so that, in contrast to nitrification/−denitrification, no C is required (Zekker et al., 2021). If the organic components in the wastewater are separated efficiently before mainstream deammonification, they can be used for energy generation in the form of biogas through anaerobic fermentation (German Association for Water, Wastewater and Waste, 2017). Since the implementation of deammonification in the mainstream of MWWTPs would result in energy savings and is thus suitable to contribute to climate-neutral and cost-efficient wastewater treatment, the process has gained more attention during the last two decades.

However, deammonification is currently used mainly in the side stream treatment of high-strength process water after sludge dewatering. The conditions prevailing there, such as a relatively uniform amount of generation, a comparatively warm temperature, and a high total N content (>1,000 gN/m^3^) with a low chemical oxygen demand: nitrogen (COD:N) ratio < 2, principally offer optimum conditions for deammonification (Lackner et al., 2014). The application of deammonification in the mainstream of MWWTPs still faces major challenges mainly due to the mainstream conditions, such as operating temperatures below 20°C, fluctuating pH values of wastewater, low strength wastewater (< 200 gNH_4_-N/m^3^) and high concentrations of organic matter or COD:N ratios (> 10). Those conditions affect the biological process of deammonification (Gustavsson et al., 2020) and complicate the implementation of deammonification in the mainstream compared to the side stream. The foremost challenge for the implementation of mainstream deammonification is the high concentration of organic substances that determine the sludge composition and thus interfere with the anammox process. To use the organic substances for biogas production, they must be separated from the wastewater *via* an additional upstream C removal stage. Another challenge is the selection of the required microorganisms, as complex bacterial interactions lead to the process being described as unstable especially at low temperatures and at high COD:N ratios.

Since the last decades, there have been many studies conducted on the determination of optimal conditions for deammonification, the effect of reactor technologies such as moving bed biofilm reactor (MBBR) and configurations such as integrated fixed-film activated sludge (IFAS), the impact of different biofilm carriers and additives such as salts and polymers for the process stability, the identification of the diversity and distribution of involved microorganisms and examination of their growth conditions (Liu et al., 2021; Feng et al., 2022; Jiang et al., 2022; Ladipo-Obasa et al., 2022; Schoepflin et al., 2022; Zhang et al., 2022). So far, most of the studies have been carried out on a laboratory scale with artificial wastewater. Only a few investigations have been carried out with real wastewater on a pilot scale or in large-scale technology (Lotti et al., 2015). Increasingly, deammonification studies have been conducted in warmer climates due to better temperature conditions in wastewater (Nsenga Kumwimba et al., 2020). A recent full-scale study on anammox under ambient temperatures and without bioaugmentation was reported at a wastewater treatment plant in China (Yuan et al., 2021). These studies however showed that mainstream deammonification is feasible at moderate temperatures and further optimization by a more dedicated design is needed for an improved N removal in mainstream wastewater treatment.

A full-scale stable mainstream deammonification was achieved at the Changi water reclamation plant in Singapore at a relatively high operational temperature of 30°C (Cao et al., 2017). However, the mainstream conditions of the MWWTPs differ largely among the climate zones of the earth. European countries like Germany have wastewater temperatures between 15°C (winter) and 25°C (summer) in their mainstreams, while south-east countries like Singapore have wastewater temperatures at 30°C that apply to none of the European countries. There are comparatively few studies among all those under mainstream conditions on pilot-scale concerning temperate zones of the earth such as ANITA™Mox and full-scale mainly at MWWTP Sjölunda in Sweden, MWWTP Zillertal in Austria, MWWTP Glarnerland in Switzerland (Wett et al., 2010; Thomson et al., 2016; Christensson et al., 2018; Stefansdottir et al., 2018; Weissenbacher and Wett, 2018). Though most of these studies proved a relevant performance of mainstream deammonification, several biological and engineering challenges were confronted (Wett et al., 2010; Thomson et al., 2016; Christensson et al., 2018; Stefansdottir et al., 2018; Weissenbacher and Wett, 2018). There are no successful engineering projects in treating low-strength municipal wastewater in temperate zones of the earth like Europe so far. Stability has not yet been achieved in effluent conditions. Long-term suppression of nitrite-oxidizing bacteria has not yet been demonstrated (Christensson et al., 2018; Stefansdottir et al., 2018). Studies like ANITA™Mox are still ongoing for the optimization of bioaugmentation. ANITA™Mox process is developed by Veolia Water Technologies as an alternative to conventional nitrification/− denitrification processes (Thomson et al., 2016). It is a pilot-scale single-stage deammonification process utilizing MBBR and IFAS technologies, being carried out in Paris. Enhanced C removal stage prior to the mainstream anammox treatment at 13–23°C was performed using a drum filter. With appropriate aeration and operational control, it was possible to achieve good effluent quality (8–15 gN/m^3^) downstream of the ANITA™Mox system without any further polishing stage for N removal. These studies are currently ongoing. Further optimization of bioaugmentation from the sidestream to the mainstream should improve robustness and final effluent quality for the mainstream application.

However, there are no studies yet focused on the adaptability of side stream deammonifying sludges for mainstream applications. The major challenge of mainstream deammonification is first of all to match the nitrogen removal rate (NRR) attained by the state of the technology, nitrification/denitrification, without compromising the performance of MWWTPs in temperate zones of earth, e.g., Germany. The performance of NRR of several deammonifying sludges (located currently in side streams) and their adaptability under mainstream conditions of MWWTPs are unknown. Therefore, the primary aim of the current study is not to find the optimum operational parameters for the microbial community of deammonification, but rather to determine the operational window of different deammonifying sludges in Germany with respect to important process parameters (temperature, pH and COD:N ratio). The determined operational window should serve as the performance indicator of the tested deammonifying sludges, in which they can perform relevant NRRs that are in the same order of magnitude as the activated sludge process, nitrification/−denitrification. Further, the operational window should serve to find out the limits of the process, at which it is largely inhibited and becomes unstable. In this regard, batch tests were carried out on a laboratory scale for various deammonifying sludges from the side stream deammonification reactors of seven MWWTPs in North Rhine-Westphalia, Germany using as inoculums of the lab-scale reactors. Of particular interest is the question of whether heterotrophic bacteria in the deammonifying sludges used to side stream conditions become more active at different COD:N ratios in wastewater.

**Figure 1:**
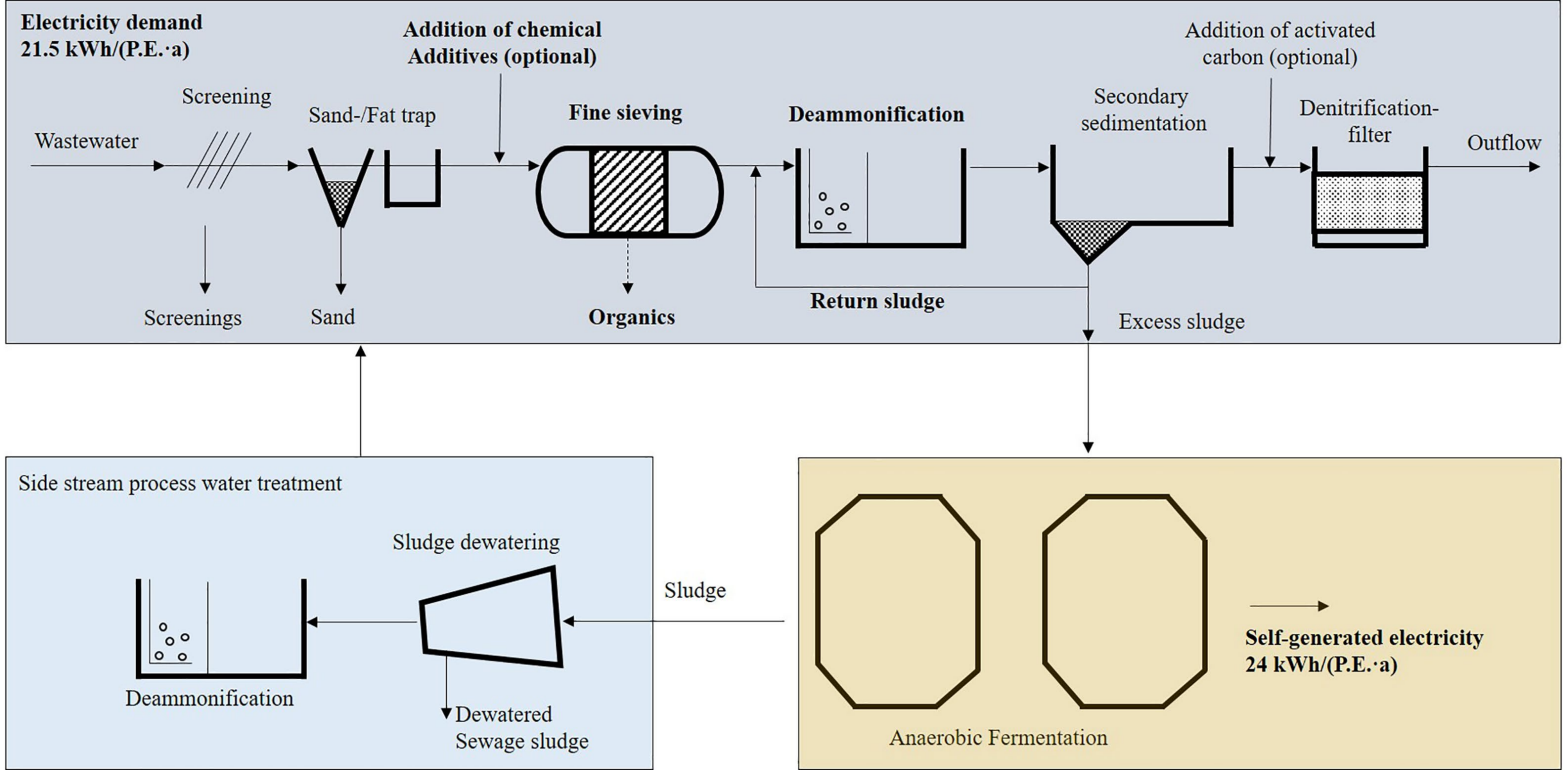
Conceptual model of a municipal wastewater treatment plant with mainstream deammonification depicting its energy saving and generating potential.

Based on the determined operational limits of tested deammonifying sludges as inoculums in laboratory experiments, it is necessary to accept and solve the process engineering challenges for the mainstream application. For this purpose, a conceptual model plant with mainstream deammonification was developed in the current study as shown in (Figure 1). Since the nitrification/−denitrification process is being used both for N and C removal in the mainstream of MWWTP and deammonification cannot eliminate C, an alternative approach for C removal was addressed in the developed model. Since the energy required for the C elimination will deduct some of the savings credited to mainstream deammonification, the energy balance of the developed model plant was evaluated with respect to electricity demand and self-generated electricity. Moreover, the sensible integration of deammonification into the overall municipal wastewater treatment process was proposed in the current study in terms of economical construction and retrofitting scenarios. The developed model plant of mainstream deammonification was compared with a conventional activated sludge plant from the perspectives of energy potential, N removal performance and economical construction. Thereby, providing a solid basis to expand the knowledge on the implementation of mainstream deammonification with possible solutions for the challenging mainstream conditions in the temperate zones of the earth.

## 2. Materials and methods

### 2.1. Laboratory experiments of mainstream deammonification

For conducting the batch tests, a Sixfors® laboratory-scale plant (Infors® HT, Switzerland) was used. The plant consisted of six parallel reactors, each equipped with a temperature sensor (pt100) and an automatic temperature controller, pH sensor (Redox-electrode, Mettler Toledo), dissolved oxygen sensor (polarographic electrode, Mettler Toledo), magnetic stirrer, aeration units, and a common U-Direct Digital Control (U-DDC) operating panel. Each reactor had a total and operating volume of 0.0006 m^3^ and 0.0005 m^3^, respectively. The “Iris” software, which connected the laboratory-scale plant to a desktop, controlled all the operational parameters including process control and data logging,−analysis, and-export.

Deammonifying sludges from the side stream of seven MWWTPs in Germany were investigated as seeding sludge or inoculum in the batch tests for N removal performance. The tested seeding sludges are denoted as MWWTP 01 to 07 in this work. Since the biomass activity dies or largely drops when the process reaches its procedural limits during the batch tests, a model plant of two-stage deammonification was set up in the lab as a backup reactor. The backup reactors, one (0.025 m^3^ reactor volume) for the partial nitritation stage and the other (0.01 m^3^ reactor volume) for the anammox stage, were individually inoculated with the respective deammonifying sludges to be studied. The partial nitritation reactor was intermittently aerated every 4 h. The backup reactors were run at an optimum temperature of 30°C to keep the biomass in the sludges active.

The six parallel reactors of the Sixfors plant for the batch tests were run as sequencing batch reactors (SBR). One SBR cycle refers to a single batch test in this study. SBR filling was divided into two steps: (i) 0.00025 m^3^ of fill volume was the respective inoculated deammonifying sludge from the backup reactors and (ii) the 0.00025 m^3^ was effluent/centrate from the sludge dewatering of large-scale regional MWWTPs in Germany. Centrate was used only to increase the ammonium (NH_4_-N) start concentration in the batch tests to match the N loads of mainstream MWW. The original composition of the studied deammonifying sludges at the time of sampling from large-scale reactors and used centrate are to be found in Table 1.

**Table 1:** The original composition of the tested side stream deammonifying sludges as mainstream inoculum and centrate from various MWWTPs.

Parameter	TN_b_ [g/m^3^]	NH_4_-N [g/m^3^]	NO_2_-N [g/m^3^]	NO_3_-N [g/m^3^]	PO_4_-P^a^ [g/m^3^]	SO_4_^2-b^ [g/m^3^]	CSB [g/m^3^]	TS^c^ [g/m^3^]	SV30 [ml/l]	pH-value [−]
Centrate 01	634	633	0.09	0.89	21.9	0	339	< 0.01	0.05	7.6
Centrate 02	554	554	0	0	25.5	87.9	178	0	0.05	7.4
MWWTP 01	410	263	5	85.3	-	-	600	2.6	-	8.2
MWWTP 02	200	138.5	14	47	-	-	250	1.25	-	6.8
MWWTP 03	1,300	583.5	500	58	-	-	-	1.42	-	6.5
MWWTP 04	35.5	14.5	1	1.1	14.7	61.9	100	2.28	300	7.8
MWWTP 05	321	262	6.4	54.7	48.7	98.4	624	3.6	1,450	7.1
MWWTP 06	450	450	0	0	-	-	193	3.69	-	7.9
MWWTP 07	285	189	6	88.1	45.8	91.1	647	1.9	150	7.4

^a^Ortho phosphor, ^b^Sulphate, ^c^Total solids.

SBRs were operated in a 12–20 h SBR cycle depending on the origin of the tested inoculum or deammonifying sludge. SBRs with the sludges of MWWTP 01–02 and 05–06 originated from a single-stage deammonification reactor and were therefore operated for 8 h of aerobic phase and 12 h of anoxic phase, in which the NH_4_-N start concentration of 150 ± 0.01 g/m^3^ was provided using centrate after sludge dewatering from a local MWWTP. SBRs with the sludges of MWWTP 03, 04, and 07 originated from the anammox reactor of a two-stage deammonification process and were therefore investigated only for an anoxic phase of 12 h, where NO_2_-N in the SBR was provided by sodium nitrite. The initial stoichiometric NO_2_-N/NH_4_-N ratio of 1.32 was kept during the batch tests for the sludges of MWWTP 03, 04, and 07. A wide range of operational parameters like temperature, initial pH, and influent COD:N ratio were tested in duplicate determination. Acetic acid was used to adjust the ratio of COD:N ratio. Each operational parameter was studied in the selected range shown in Table 2 while keeping the other two parameters constant.

**Table 2:** Operational parameters tested in laboratory batch tests.

Parameter	Temperature [°C]	pH-value [−]	COD:N ratio [−]
Batch tests of temperature	**8–45**	7.5–7.8	≤ 1.5
Batch tests of pH-value	20–30	**4–10.5**	≤ 1.5
Batch tests of COD:N ratio	20–30	7.5–7.8	**1.5–6**

Wastewater samples at the start and end of the aeration phase and the end of every batch test were collected. The respective concentrations of N compounds in the samples were examined for the confirmation of the metabolic pathway of the deammonification process. The concentration of total N (LCK 338, EN ISO 11905-1), NO_2_-N (LCK 342, DIN 38405 D10), PO_4_-P (LCK 350, DIN EN 6878 / D11), SO_4_^2−^ (LCK 153), and COD (LCK 114, DIN 38409-H41-H44) were measured photometrically using cuvette test kits (Hach Lange®, Germany). The error of proximity in the measurement was assumed to be 8%. The concentration of NH_4_-N (Art. Nr. 100,683) and nitrate (NO_3_-N, Art. Nr. 101,842) were analyzed spectro-photometrically using test kits (Merck®, Germany) according to EN ISO 9001. TS in the deammonifying sludges were determined according to DIN EN 12,880. Sludge Volume 30 is measured by a settling test according to DIN EN 14702–1. The pH of the sludges and centrate after sampling from the MWWTPs was measured using GE 117 pH electrode (Greisinger® 600,770).

### 2.2. Description of conventional activated sludge plant with upstream denitrification

To be able to find out the suitability of tested deammonifying sludges in the current work for mainstream application at the developed model plant in section 2.3 the standard or minimum requirement of NRR at conventional MWWTP must be determined. The standard NRR is nothing but the NRR achieved by the activated sludge process, nitrification/−denitrification, in the mainstream of conventional MWWTP. Furthermore, the tested deammonifying sludges should meet the similar requirement of reactor volume size as the nitrification/−denitrification process at conventional MWWTP for mainstream suitability. Therefore, the NRR of the deammonification process is from here on referred to as volumetric nitrogen removal rate (VNRR) in the current work, corresponding to the reactor volume.

For this purpose, a sample conventional MWWTP was designed for a size of 30,000 P.E. using the dimensioning software Design2Treat® (Institut für Siedlungswasserwirtschaft (ISA) der RWTH Aachen, 2022) that corresponds to a size class of 4 according to the classification of MWWTPs in Germany. As a rule, the division into size classes is based on the capacity of population equivalent (P.E.). Size class of 4 represents the capacity of 10,001–100,000 P.E. The design was based on the assumption of a standard conventional MWWTP in Germany, consisting of three main treatment stages: the preliminary treatment for removal of coarse solids through mechanical screenings and grit chambers; the primary treatment for removal of settleable organic and inorganic suspended solids through sedimentation; the secondary treatment for removal of nutrients and organic substances biologically through activated sludge process. The secondary treatment uses nitrification/−denitrification for N and C removal and chemical precipitation for phosphorous removal. The subsequent secondary sedimentation ensures the return of activated sludge into the biological reactors. The conventional design of the plant provides a required specific reactor volume size of approx. 0.173 m^3^/(P.E.) for nitrification and denitrification and a specific electricity demand of 35 kWh/(P.E.·a) at a design temperature of 12°C. For the design of the plant, it was assumed that the values for a size class 4 of MWWTP according to Annex 1 of the Wastewater ordinance on requirements for the discharge of wastewater into Waterbodies by the Federal Ministry for the Environment, Nature Conservation and Nuclear Safety, Germany (2004) apply as monitoring values. The main input data, assumptions and requirements for the design are summarized in Table 3. Table 4 describes the volume capacity of each treatment stage, resulting from Design2Treat. Further assumptions and characteristics of the designed MWWTP can be found in Supplementary material S1.

**Table 3:** Basis of assessment for the conventional wastewater treatment for 30,000 P.E.

Hydraulics			Loads (DWA A 131)			Assumptions		
Parameter	unit	value	Parameter	unit	value	Parameter	unit	value
Specific wastewater accumulation	m^3^/(P.E.·d)	0.125	BSB_5_	kg/d	1,800	Design temperature	°C	12
Dry weather inflow	m^3^/h	352	TS_0_ (FS^a^)	kg/d	2,100	Mean flow time in primary sedimentation	h	1
Mixed water inflow	m^3^/h	664	NH_4_-N	kg/d	210	Fluctuation factor^b^	-	2
External water inflow	m^3^/d	938	N_org_	kg/d	120	f_C_^c^	-	1.5
Daily inflow	m^3^/d	4,688	P_total_	kg/d	45	f_N_^d^	-	1.2
-		-	-	-	-	Sludge volume index	l/kg	120

^A^filterable substances, ^b^According to university group approach, ^c^Shock load factor for carbon, ^d^Shock load factor for N.

**Table 4:** Characteristics of the treatment stages of conventional MWWTP for 30,000 P.E.

Parameter	Unit	Primary sedimentation	Nitrification	Denitrification	Secondary sedimentation
Volume	m^3^	350	3,285	1,905	2,550
Surface	m^2^	-	-	-	541

### 2.3. Description of the developed MWWTP with mainstream deammonification

Based on the previous demonstrations and full-scale studies on deammonification in the mainstream of MWWTPs, the results of semi-technical trials for COD removal by Kaleß and Pinnekamp (2017) and the laboratory results of VNNRs from section 3.1 in the current study, a concept of the MWWTP with mainstream deammonification was proposed in this work. Regarding the VNRR, the plant model was designed to be able to fulfill the same requirements as the conventional activated sludge plant dimensioned in section 2.2. As with the conventional activated sludge plant, the assumed prerequisite for the mainstream deammonification plant model was, that the wastewater is mechanically pre-treated *via* a screening step and a grit chamber for sand and grease. It is followed by the combined chemical precipitation of phosphorus and dissolved organics using 0.03 kg/m^3^ iron and 5 ppm polymer and a C removal stage that uses an ultra-fine screen with a mesh size of 0.3 mm in the form of a drum filter as shown in Figure 1. This way 70–90% COD is reduced from the wastewater reaching a possible COD:N ratio of 2.5:1 prior to the mainstream deammonification stage according to Kaleß and Pinnekamp (2017).

According to the work-sheet DWA-M 349 of the German Association for Water, Wastewater and Waste (2019), the estimation of the required reactor volume of a single-stage reactor for mainstream deammonification was carried out based on the VNRR required for an MWWTP of size class of 4. If it is also assumed that 1 gN/(P.E.·d) is eliminated from the overall inhabitant-specific load of 11 gN/(P.E.·d) in the primary clarification, approximately 7.5 gN/(P.E.·d) must be eliminated in the biological stage at a N elimination rate of 75% to meet the stringent discharge limits of N according to wastewater ordinance in Germany. Without taking into account inlet fluctuations and shock loads, this corresponds to a VNRR of at least 50 gN/(m^3^∙d). In the laboratory batch tests of section 2.1 a VNRR of at least 50 gN/(m^3^∙d) was achievable by various deammonifying sludges under mainstream conditions (8–20°C and pH 6–8, NH_4_-N start conc. 150 ± 0.01 g/m^3^). For a total N elimination capacity of 83% as stated in the latest 33^rd^ DWA performance record of MWWTPs by the German Association for Water, Wastewater and Waste (2020), the required reactor volume for mainstream deammonification resulted as listed in Table 5. Thus, the inhabitant-specific reactor volume for mainstream deammonification is in the order of magnitude for the conventional activated sludge process depending on the proportion of N_organic_ separated in the ultra-fine screening at the C removal stage. However, the dimensioning of the conventional activated sludge plant according to Deutsche Vereinigung Für Wasserwirtschaft, Abwasser Und Abfall EV (2016) includes surcharges, which reflect dynamic effects in the course of loads. These uncertainties are not included in the estimation of the required reactor volume for mainstream deammonification.

**Table 5:** Required reactor volume per inhabitant for mainstream deammonification at different efficiencies of the pretreatment.

Parameter	Unit	Value		
The efficiency of ultra-fine screening at C removal stage	%	50	70	90^a^
N_organic_ retained in the ultra-fine screening	g/(P.E.∙d)	2	2.8	3.6
Required reactor volume at a standard VNRR of 50 gN/(m^3^∙d)	m^3^/P.E.	0.149	0.132	0.115

^a^With chemical pre-precipitation.

For the secondary sedimentation, a smaller volume of the reactor would be expected as the settling properties of the deammonifying sludge are better than those of conventional activated sludge. The sedimented sludge from the secondary clarification is assumed to be carried back to the partial nitritation stage of deammonification as return sludge or discharged as excess sludge for sludge treatment. Based on the reports of demonstrational and large-scale plants for deammonification in the mainstream, a residual load of COD and NO_3_-N should be removed in the effluent of the deammonification stage. The non-filterable part of the COD could pass the ultra-fine screen almost unaffected without chemical additives. The proportion of the conversion of this organic load is unknown in mainstream deammonification. In this regard, the entire plant is to be equipped with a biologically activated filter downstream of the secondary sedimentation to denitrify the NO_3_-N using the available C and simultaneous COD removal. The dimensioning of the filter stage is based on the maximum filter velocity of 15 m/h at mixed water inflow. This results in a required filter area of 44.3 m^2^. In dry weather conditions, the filter velocity can be 7.95 m/h and thus within a normal range. Three filters with a width of 4 m and a length of 7 m should be provided for the reliable operation of the plant. To be able to denitrify the NO_3_-N in case of a lack of available C, a storage and dosing station for acetic acid should be provided.

### 2.4. Assumptions and conditions applied for the comparison of the concepts

The developed concept of mainstream deammonification from section 2.3 was compared with the conventional activated sludge model from section 2.2 in terms of VNRR, energy potential, and economical construction/retrofitting perspectives.

The VNRR is defined as the amount of total bound N eliminated per cubic meter of reactor volume in a day [gN/(m^3^·d)]. The VNRR of deammonification was experimentally determined for various deammonifying sludges under mainstream conditions according to section 2.1. As per the daily loads of N and reactor volume in the mainstream at the conventional MWWTP listed in Table 3 and Tables 4, a 63.5 gN/m^3^_reactor volume_ is subjected to the mainstream nitrification/−denitrification. In this regard, the VNRR of 50 gN/(m^3^∙d) is set as the design basis for mainstream deammonification to be achieved in the mainstream based on the laboratory results of various deammonifying sludges.

The data for the energy demand and self-generated electricity of conventional MWWTP with activated sludge process was extracted from the results of Design2treat software and the yearly reports of the German Association for Water, Wastewater and Waste (2020) such as the 33^rd^ DWA performance record of MWWTPs. The data for the energy demand and self-generated electricity of model MWWTP with mainstream deammonification was calculated based on the results of Design2treat software, Kaleß and Pinnekamp (2017) and the yearly reports of the German Association for Water, Wastewater, and Waste.

The treatment stages such as screening plant, grit/grease trap, anaerobic sludge treatment, sewage gas storage, treatment and utilization, and side stream process water treatment are common for both concepts and therefore not included in the estimation of investment. Table 6 describes the assumed characteristical sizes of the treatment steps involved in the estimation of cost.

**Table 6:** Estimated volumes for the new construction of the compared processes.

Process step	Activated sludge process with upstream denitrification		Mainstream deammonification	
	Unit	Characteristic size	Unit	Characteristic size
Primary sedimentation	m^3^	352		**-**
Ultra-fine sieve, enclosed, two-line, (mixed water inflow)		-	m^3^/h	664
Activated sludge reactor, two-line		5,840	m^3^	5,840
Secondary sedimentation		2,550	m^3^	2,550
Biological activated filter area		-	m^2^	3 × 21

In addition, the possibility of converting existing conventional MWWTPs to mainstream deammonification was examined. Since almost all the inhabitants in Germany are connected to MWWTPs, the most important categories considered for retrofitting were: the possibility of using existing infrastructure/reactor volumes; additional machine technology to be purchased; additional measurement, control, and regulation equipment to be purchased.

## 3. Results and discussion

### 3.1. The VNRR of various deammonifying sludges

**Figure 2:**
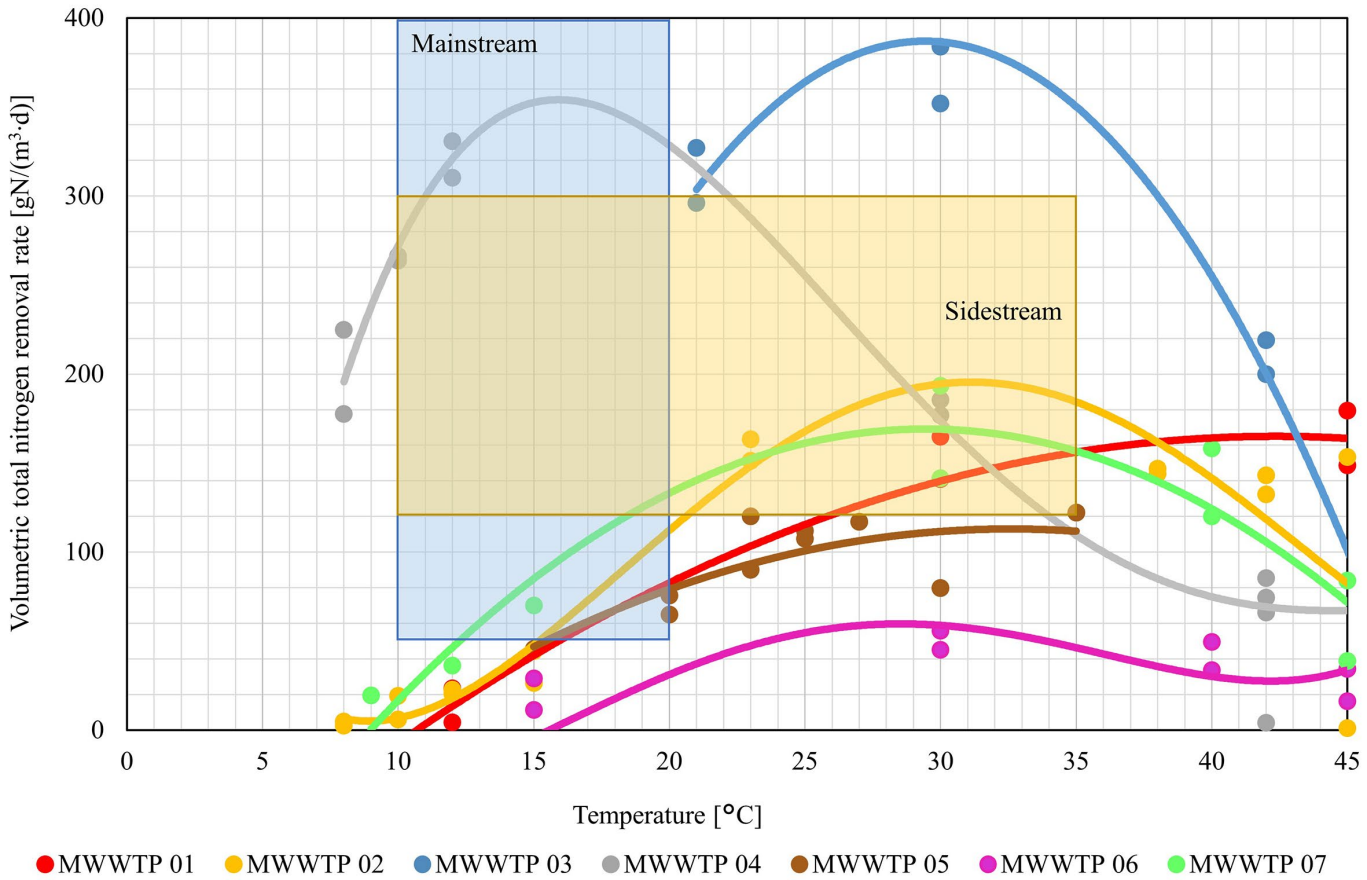
The volumetric nitrogen removal rate of the deammonification process for various deammonifying sludges as a function of temperature.

Figure 2 shows the VNRR by the tested deammonifying sludges during the deammonification process at different temperatures. The elimination was related to the VNRR, both the N_inorganic_, a sum of NH_4_-N, NO_2_-N, and NO_3_-N, and N_organic_. Among all the parameters prevailing in the mainstream, Temperature is particularly relevant for the deammonification process. The temperatures between 30 and 35°C are most favorable for deammonification (Wang et al., 2018; Le et al., 2022). For the tested deammonifying sludges, the VNRR range from 3.8 to 220.8 gN/(m^3^∙d) in large-scale side stream treatment at warm conditions (20–30°C). In contrast, VNRRs up to 384 gN/(m^3^∙d) were achieved by the deammonifying sludges in the lab-scale at warm temperatures (30°C) in the current study, e.g., MWWTP 03. Le et al. (2022) reported a stable VNRR of 250 ± 3 gN/(m^3^∙d) at 32–35°C in lab trials using bio-carrier reactor technology. In a pilot study by Huang et al. (2023) using fluidized membrane bio-reactor technology, the VNRRs accounted for 60.6 ± 13.3 gN/(m^3^∙d) at 28–30°C with the effluent TN below 10 gN/m^3^ at the HRT of 8 h. VNRRs 384 gN/(m^3^∙d) of deammonification at 30°C of the present work achieved higher efficiencies using SBR technology than other reactor technologies in literature. Nevertheless, the results of Huang et al. (2023) should not be seen as a direct comparison to the current study as the VNRRs represented the pilot-scale study. The anammox activity in the pilot-scale systems, comparable to lab-scale systems, could be variable over time in response to the temperature (Hausherr et al., 2021).

Figure 2 illustrates clearly that the biological activity decreased in particularly cold (< 15°C) and very warm (> 40°C) environments. Genera belonging to bacterial phylum Planctomycetes mediate the anammox processes, whose proliferation (μ_max_ of 0.049 d^−1^ at 37°C; 0.17 d^−1^ at 22°C) is slow and strongly influenced by temperatures (Ali et al., 2015; Zhang et al., 2017). Sudden changes in temperatures can easily reduce anammox activity (Tomaszewski et al., 2017). Recent reports indicated that the anammox reaction of these strains is recommended to carry out at the optimum temperature between 30 and 37°C for stable N removal. However, these temperatures do not apply to the mainstream conditions in the temperature zones of the earth like Germany (Le et al., 2022).

Reduced activity of 50 gN/(m^3^∙d) or less was observed above 40°C compared to a VNRR of up to 384 gN/(m^3^∙d) at the optimum temperature (30°C). Isanta et al. (2015) reported that the anammox community in his lab trials was shifted in a week from *C. Kuenenia* to *C. Brocadia anammoxidans* after the temperature increased from 35C to 46°C, and N removal reduced to 1/10 [approximately 300 gN/(m^3^∙d)]. Similarly, the VNRRs in the current study started to decrease already from 35°C but reduced to 1/8 of VNRR occurred at 30°C. Moreover. a similar magnitude of VNRR was observed at temperatures below 30°C. The efficiency of VNRR was reduced to 80% [64 gN/(m^3^∙d)] when the temperatures dropped from 30°C to 20°C. Five of the tested deammonifying sludges (MWWTP 01, 02, 04, 05, 07) provided VNRRs from 50 to 336 gN/(m^3^∙d) between 10 and 20°C in the mainstream operational window (blue window on Figure 2). This is comparable with the reports of Le et al. (2022) in which a reduced NRR of 100 gN/(m^3^∙d) resulted when the temperature abruptly dropped to 18°C. In the current study, the VNRR tended to decrease for most of the MWWTPs and reached removal rates below 50 gN/(m^3^∙d) by 15°C. The exception was the deammonifying sludge from MWWTP 04, which achieved the highest VNRR of 312 gN/(m^3^∙d) at 12°C and did not lose performance very rapidly below 15°C. This behavior of the deammonification system is in line with the study of Hausherr et al. (2021). The anammox systems can be well adapted to seasonal temperature variations sustaining the target VNRR. Hausherr et al. (2021) reported a VNRR of 200 gN/(m^3^∙d) down to temperatures of 13°C and 260 ± 83 gN/(m^3^∙d) were removed on average after the start-up.

**Figure 3:**
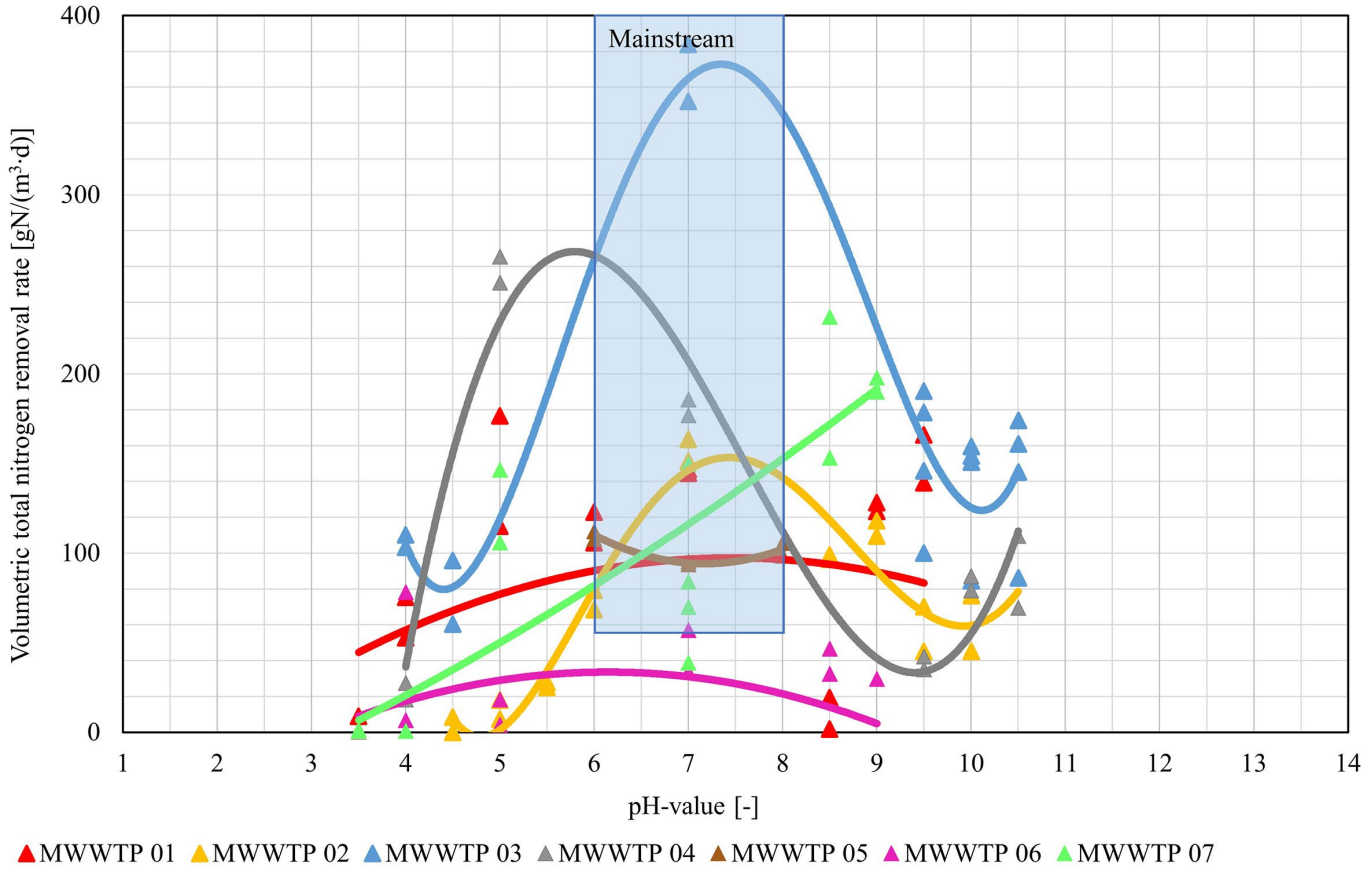
The volumetric nitrogen removal rate of the deammonification process for various deammonifying sludges as a function of the initial pH value at the start of the batch test.

Figure 3 shows the VNRR of various deammonifying sludges at different initial pH values. The equilibrium between ammonium and ammonia is dependent on pH (Montag, 2008). Therefore, especially at higher pH values, stripping processes can occur more frequently. At a pH above 9, about 45% of the N would be present as ammonia and would outgas (stripping effect) over time. This proportion could not be detected in the N analyzes and could therefore be misinterpreted as biological elimination. Figure 3 shows a decrease in VNRRs at higher pH values. At pH values >9, the VNRR was about 50% smaller than the maximum value observed at pH 7. The performance at pH 10.5 was only about 25% of the maximum value observed at pH 7. A complete reduction of N conversion could not be observed despite pH values of up to 10.5. While stripping seems to occur at pH > 9, no relevant stripping effects are to be expected at pH values less than 9. Figure 3 shows that all of the tested deammonifying sludges except MWWTP 07 achieved a VNRR between 50 and 384 gN/(m^3^∙d) in the relevant pH value range of 6–8 occurring in mainstream treatment (blue window in Figure 3). MWWTP 03 and 05 even showed a VNRR of at least 50 gN/(m^3^∙d) at and below pH 5. The VNRR of all the investigated deammonifying sludges reached its minimum performance at pH values by 4.

**Figure 4:**
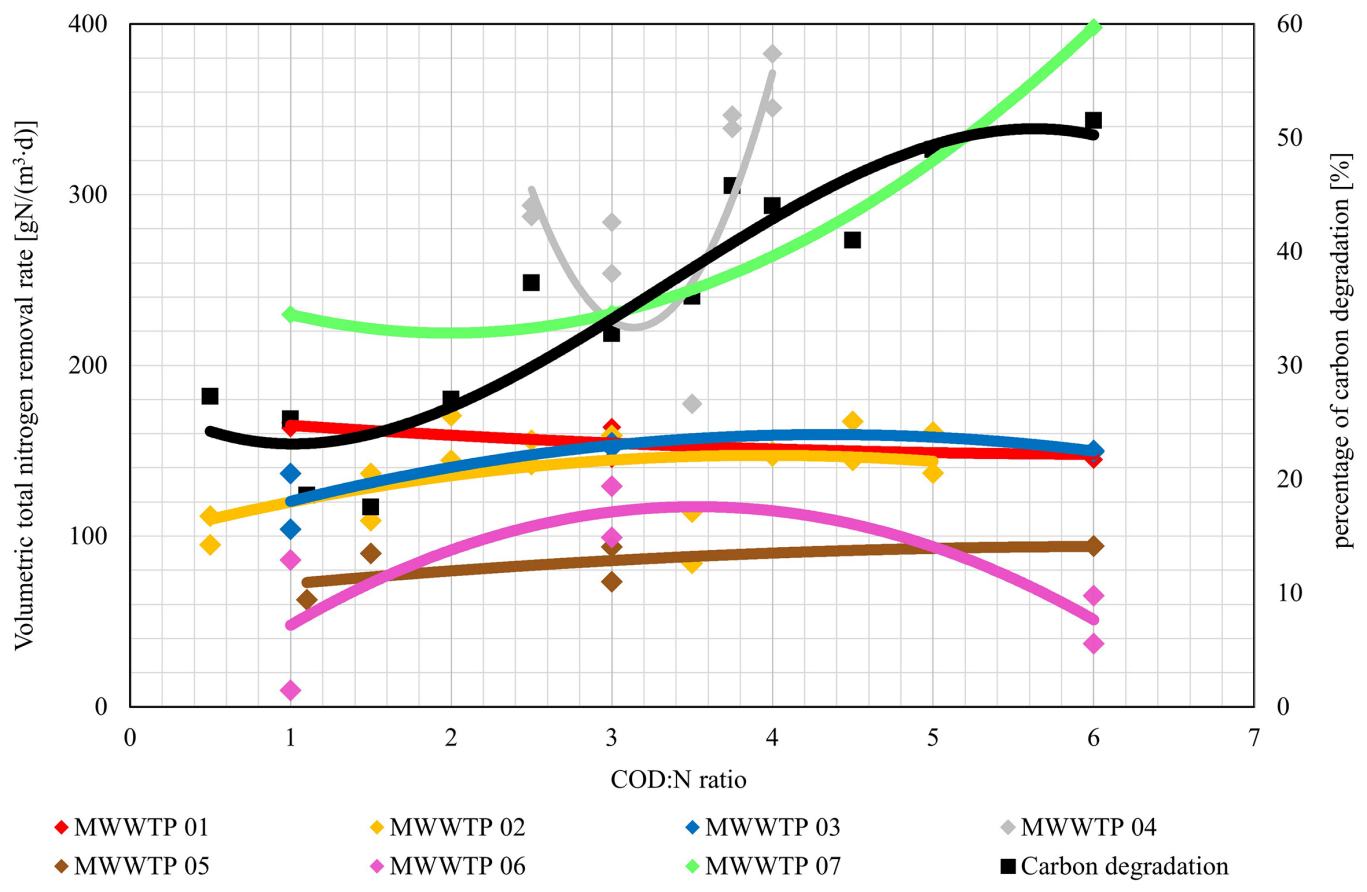
The volumetric nitrogen removal rate of the deammonification process for various deammonifying sludges as a function of COD:N ratio.

Figure 4 shows the VNRR of the deammonification process for various deammonifying sludges as a function of COD:N ratio. Stoichiometrically, no C is required for the deammonification process. However, the higher the C content, the greater the advantages for the growth of heterotrophic bacteria, which displace the deammonifying biomass (Kartal et al., 2007; Xu et al., 2015). As a result, the VNRR in relation to total N does not drop even at higher COD:N ratios, but the metabolism changes. To be able to assess whether the measured N elimination is based on deammonification or nitrification/denitrification, C consumption was used as an additional parameter.

The C degradation, black curve, in Figure 4 denotes the mean consumption of COD that occurred during the laboratory experiments of all of the tested deammonifying sludges at respective COD:N ratios. From the C degradation in Figure 4, the hypothesis of displacement of deammonification by denitrification at higher COD:N ratios can be confirmed. At COD:N ratios up to 2, the C degradation increased to about 30%, while at a COD:N ratio of 6 it was 50%. As the C availability was further increased in the reactor system, the C degradation continued to increase. This reveals that at a higher COD:N ratio, heterotrophic degradation and denitrification occurred and N elimination *via* this metabolic pathway became more dominant. This leads to the conclusion, that heterotrophic degradation and denitrification occurred at COD:N ratios >2 in the current study. As a result, the VNRR does not drop even at higher COD:N ratios, but the metabolism of the biomass adjusts. Moreover, N elimination *via* the changed metabolic pathway became more efficient than *via* deammonification alone. The effluent in the mainstream at MWWTPs is characterized by a high COD:N ratio between 10 and 12, while deammonification usually is implemented on large-scale in the side stream only at COD:N ratio < 2.0. The experiments in the current study showed that all of the tested deammonifying sludges achieved the VNRR of at least 50 gN/(m^3^∙d) at COD:N ratio ≤ 2.

In a study by Azari et al. (2020), 90% of N elimination occurred at a COD:N ratio of 2.23 when the SBRs with IFAS configuration were operated at 23°C and a pH value of 6.7 to treat the synthetic wastewater. During this period, aerobic ammonium oxidizing bacteria (AOB, responsible for partial nitritation) doubled in 7 to 8 h, while nitrite-oxidizing bacteria (NOB, nitrification to NO_3_-N) doubled in 10 to 13 h. Anammox activity decreased sharply at COD concentrations greater than 99.7 g/m^3^. Similarly, in the case of MWWTP 05 in the present study, NO_2_-N and NH_4_-N degradation was not affected during the anammox phase when the COD concentration in the SBRs was lower, i.e., at 89 g/m^3^ and COD:N ratio of <2. Further increase of COD concentration > 200 g/m^3^ (COD:N ratio of >2) in the wastewater affected the NO_2_-N and NH_4_-N degradation partially. At an increased COD concentration > 550 g/m^3^ (COD:N ratio of 6), a nearly unknown percentage of deammonification was replaced by other metabolic processes such as nitrification/−denitrification. Similarly, the study by Azari et al. (2020) failed to demonstrate that the reactor is exclusively controlled by mainstream deammonification and not by simultaneous partial nitritation, anammox, and denitrification (SNAD) since heterotrophic denitrifiers could also play a role in the presence of COD. COD provides substrates for heterotrophic bacteria that compete with AOB for oxygen. A lack of NO_2_-N then affects anammox activity. Anammox bacteria (0.0027 h^−1^) cannot compete with denitrifying heterotrophic bacteria (0.062–0.108 h^−1^) in terms of growth rate (Musabyimana, 2008; Woo et al., 2022). As a result, the sludge composition in the reactor changes in favor of the denitrifiers. Instead of deammonification, nitritation and denitrification (NO_2_-N shunt) can then become the dominant process for such a system. For example, the denitrification rate in an anammox-based system can increase from 0 to 13.8% by increasing the COD:N ratio from 0 to 4 (Zhang et al., 2015). In the study by Azari et al. (2020), the adverse effects of gradually increasing the COD:N ratio > 2.0 resulted in a decrease in N removal efficiency to about 15%.

The results in the present study show that the heterotrophic bacteria become active upon increasing COD supply even though the tested deammonification sludges were only exposed to low COD:N conditions in the side stream for a long period. Therefore, a C removal stage upstream to the deammonification is essential for large-scale mainstream implementation. This can be realized using combined chemical precipitation of phosphorus and dissolved organics combined with an ultra-fine sieve for C removal prior to deammonifIcation as proposed in the developed plant model (section 2.3). A COD:N ratio of 2.5 could be reached in the effluent of C removal stage according to Kaleß and Pinnekamp (2017), which is a desirable ratio for mainstream deammonification.

### 3.2. Comparison of the concepts

#### 3.2.1. Energy potential

A few recent studies reported conceptual models of mainstream deammonification such as a three-stage deammonification process (partial nitritation/anammox reactor, acidic nitritation reactor, and anammox reactor) by implementing upstream C removal using high-rate activated sludge system prior to partial nitritation/anammox reactor in the mainstream. The acidic nitritation reactor is to serve efficient nitrite (NO_2_-N) production for the downstream anammox reactor (Cao et al., 2022; Wang et al., 2022). These concepts targeted the mainstream Anammox by integrating side stream treatment and reconfiguring the anammox reactor systems. However, these studies lack the estimation of the energy demand through the implementation of designed conceptual models with mainstream deammonification.

Based on the stoichiometry, the specific oxygen demand required for the complete oxidation of NH_4_^+^ to NO_3_^−^ during the nitrification/denitrification process is about 4.57 kg O_2_/kg N. It includes 3.43 kg O_2_/kg N for NO_2_^−^ production and a further 1.14 kg O_2_/kg N for NO_3_^−^ production (Nifong et al., 2013). Practically on a large scale, the mainstream nitrification/denitrification process requires 3.5 to 5.7 kWh/kgN_eliminated_ (Hartwig, 2007). In contrast, the deammonification process requires 1.9 kg O_2_/kg NH_4_^+^ during partial nitritation for the NO_2_^−^ conversion. This corresponds to a requirement of only 40% oxygen compared to nitrification/denitrification (Lucy Pugh, 2017). The associated lower energy requirement has been confirmed over the last decades on the large-scale implementation of deammonification in the side stream. At high temperatures above 20°C, which are unfavorable for the efficiency of oxygen transfer, deammonification requires 1.25 to 1.875 kWh/kgN_eliminated_ (O’Shaughnessy et al., 2011; Grömping and Schäpers, 2018)_._

The conventional MWWTP used in the current study yields an average resident-specific electricity demand of 23.3 kWh/(P.E.∙a) for oxygen supply during the upstream denitrification step in the single-stage activated sludge process. In total, a demand of 35 kWh/(P.E.∙a) is assumed for the operation of the MWWTP at a design temperature of 12°C. This value corresponds in magnitude to the 33^rd^ DWA performance record of MWWTP reported by the German Association for Water, Wastewater and Waste (2020), in which an average specific electricity consumption of 31.3 kWh/(P.E.∙a) is stated for the year 2020 for MWWTP of size class 4.

In the case of mainstream deammonification, a significant reduction in energy demand can be expected for N elimination. Assuming the energy requirement for mainstream deammonification of 40% (analog to oxygen demand) of the conventional activated sludge process with upstream denitrification, the energy demand for mainstream deammonification corresponds to 9.3 kWh/(P.E.∙a) for the designed size of MWWTP in this study. According to Kaleß and Pinnekamp (2017), the additional expenditure for the operation of the ultra-fine screens for upstream C removal can be estimated as 0.5 kWh/(P.E.∙a). The rest of the treatment stages such as mechanical treatment, secondary clarification, etc., do not change in both the conventional as well as the developed MWWTP with mainstream deammonification and so the respective remaining electricity demand of 11.7 kWh/(P.E.∙a). Overall, it results in a savings potential of 13.5 kWh/(P.E.∙a) of electricity for the designed MWWTP of size class 4 with mainstream deammonification. Extrapolated to the assumed concept size of 30,000 P.E., it corresponds to a saving of 405 MWh/a. A similar order of magnitude can be determined if the values of Hartwig (2007) are applied to the evaluation of energy demand. Assuming a specific energy demand of 5.7 kWh/kgN_eliminated_ and an N elimination efficiency of 83% according to Hartwig (2007), it results in a savings potential of 327 MWh/a.

**Figure 5:**
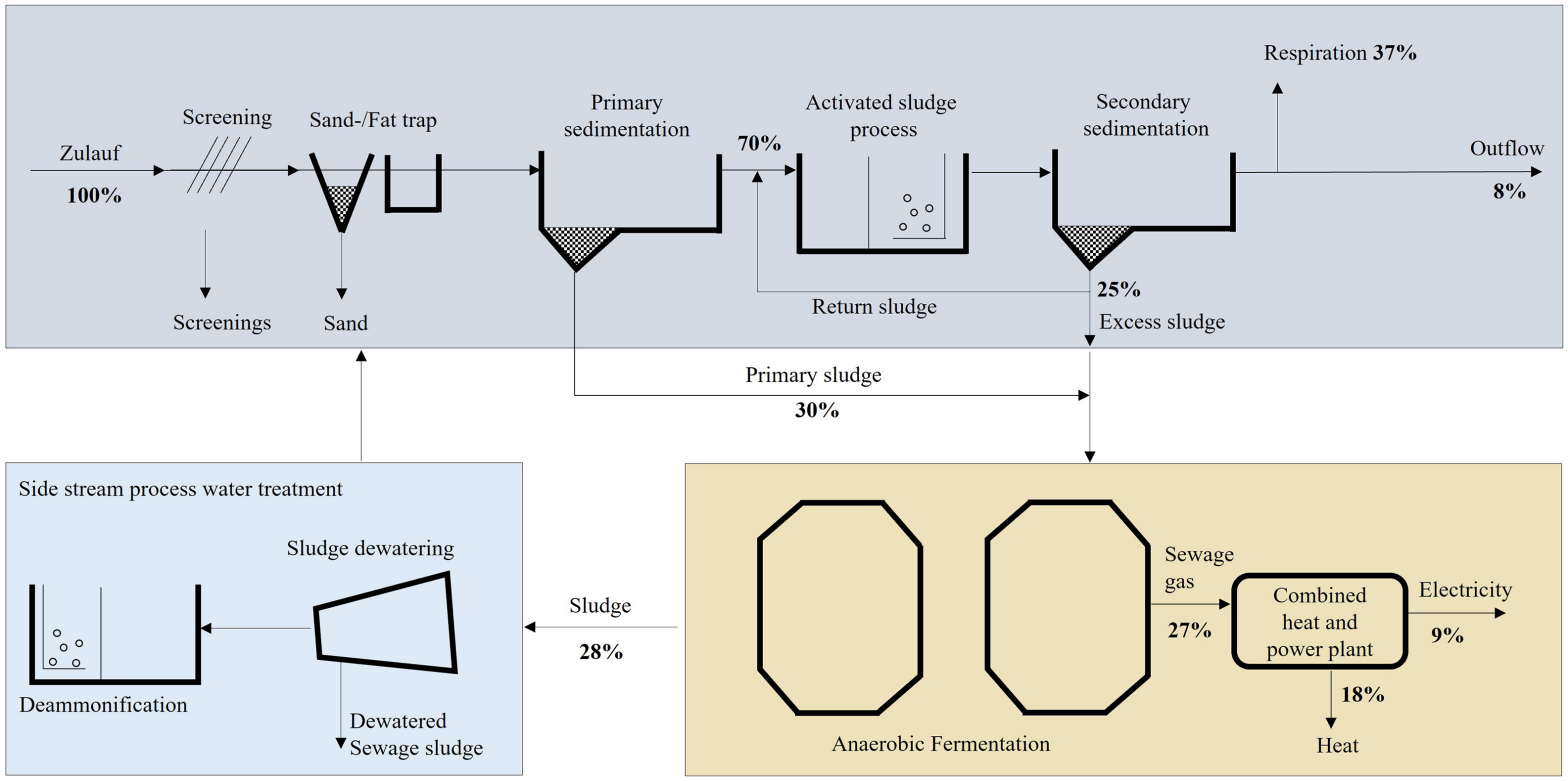
Availability of COD fractions (%) at respective treatment stages in the conventional wastewater treatment plant (modified version of Kaleß and Pinnekamp (2017)).

Furthermore, the autotrophic metabolism of deammonification does not require organic C for N removal, whereas the process of denitrification degrades organic C in the order of 5 kg COD/kg NO_3_-N (Deutsche Vereinigung Für Wasserwirtschaft, Abwasser Und Abfall EV, 2016). Due to deammonification, the unused chemical energy bound in the C can be used for sewage gas generation instead of denitrification, resulting in higher gas and thus electricity production (O’Shaughnessy et al., 2011; Izadi et al., 2021). In principle, the C in wastewater has further potential to be utilized for other purposes, such as the production of biopolymers (Valentino et al., 2017). The chemical energy content that can be used for sewage gas production in municipal wastewater is mainly present in the form of organic constituents, which are measured by the sum parameter COD. Kaleß and Pinnekamp (2017) evaluated the residual COD fractions available at respective treatment steps in the case of conventional single-stage activated sludge plant as shown in Figure 5.

**Figure 6:**
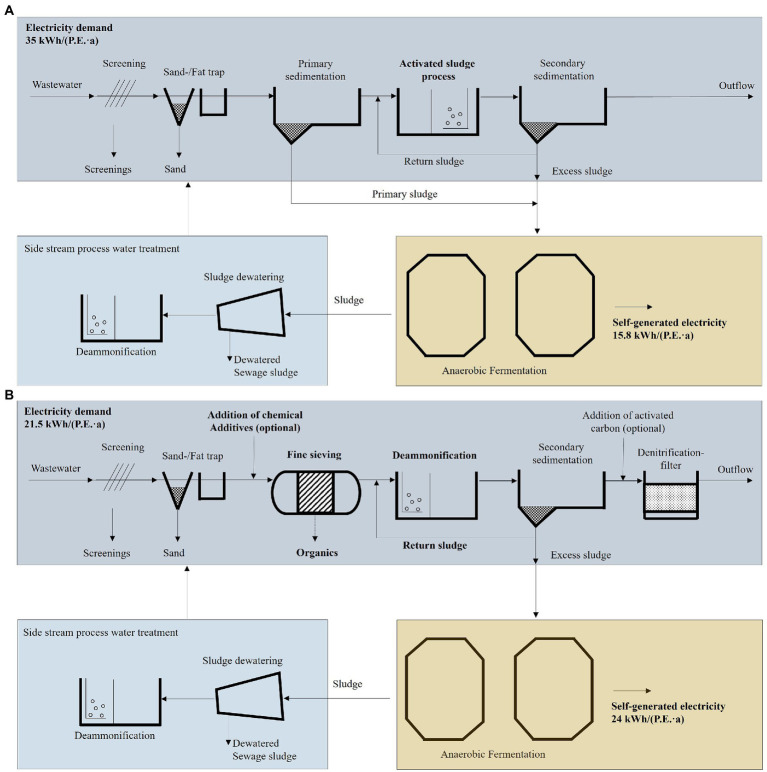
Comparison of the **(A)** conventional MWWTP with activated sludge process and **(B)** developed model MWWTP with mainstream deammonification in terms of energy demand and self-generated electricity.

Based on the report 28^th^ DWA performance record of MWWTPs by Deutsche Vereinigung Für Wasserwirtschaft, Abwasser Und Abfall EV (2015) and Kaleß and Pinnekamp (2017) stated the generated power by MWWTPs with anaerobic sludge stabilization for size class 4 and 5 as 13.5 kWh/(P.E.∙a) and 18.2 kWh/(P.E.∙a) respectively. Since the 33^rd^ DWA performance record of MWWTPs averages the degree of generated power to all the MWWTPs in Germany and additionally includes MWWTPs without sewage gas generation and its utilization, the determined degree of generated power in practice is currently approx. 36% of the size class 4 and 5, corresponding to 11,4 kWh/(P.E.∙a) (German Association for Water, Wastewater and Waste, 2020). If the generated power is only assigned to size classes 4 and 5, this value increases to 12.5 kWh/(P.E.∙a). These values are significantly lower than those given by Kaleß and Pinnekamp (2017), which can be explained by the fact that not all MWWTPs in size classes 4 and 5 are equipped with anaerobic sludge treatment. Own calculations of the energy recovery potential by Kaleß and Pinnekamp (2017) resulted in a value of 15.8 kWh/(P.E.∙a) for the conventional activated sludge stage. Based on a simulation of the effects of ultra-fine screening at the C removal stage, the average specific power generation by an MWWTP was determined as 18.2 kWh/(P.E.∙a). An amount of 19.9 kWh/(P.E.∙a) is mentioned for chemical precipitation at the C removal stage. An estimation for the combination of ultra-fine screening with the addition of chemical additives proposed in the developed concept of mainstream deammonification in the current study was not reported by Kaleß and Pinnekamp (2017). Assuming a constant availability of the COD fractions, an increase of the COD retention in the pre-treatment to 80% would result in a potential power generation of more than 24 kWh/(P.E.∙a). This calculated value matches the estimation of Siegrist et al. (2008) and Grömping and Schäpers (2018). It results in a savings potential of 8.2 kWh/(P.E.∙a) of electricity for the designed MWWTP of size class 4 with mainstream deammonification (Siegrist et al., 2008). Using these results, a comparison between the conventional activated sludge plant and the developed model plant with mainstream deammonification is shown in Figure 6 in terms of energy demand and self-generated electricity.

In the case of sewage gas power generation at the MWWTP site, the waste heat can be used for heating purposes during the plant operation following the state of the art, especially in cold seasons. However, the potential to heat the wastewater stream using the surplus waste heat is almost negligible. If a specific wastewater volume of 0.125 m^3^/(P.E.∙d), an external water content of 25% of the wastewater, and a waste heat volume of 50 kWh/(P.E.∙a) are assumed, the wastewater temperature could be increased by only 0.75°C. The potential for heating the wastewater in the mainstream to bring the temperatures closer to the side stream conditions is therefore not sufficient.

#### 3.2.2. Estimation of cost

##### 3.2.2.1. New construction of the MWWTPs

**Figure 7:**
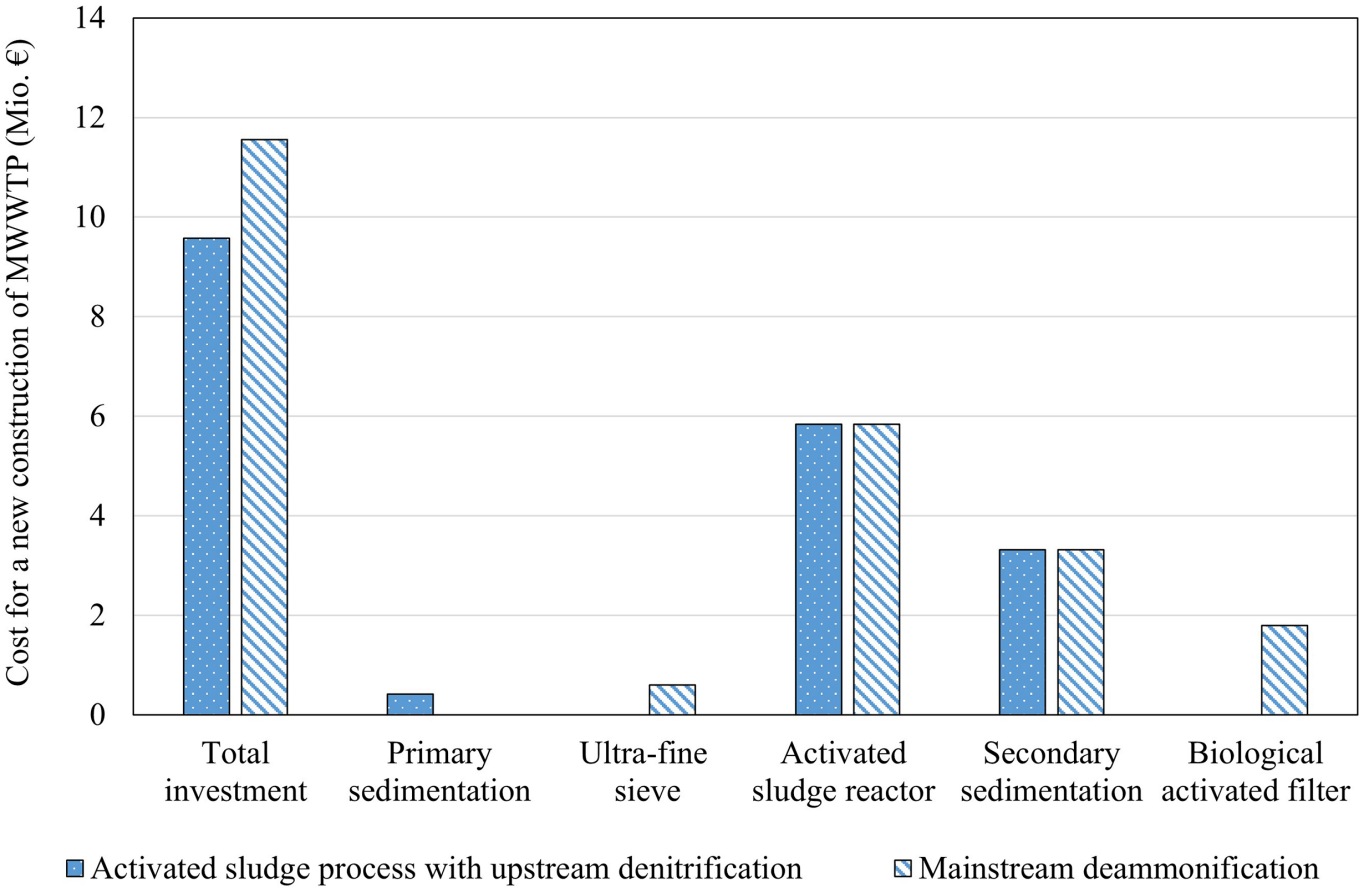
Cost estimation (net value) for the new construction of the conventional single-stage activated sludge plant with upstream denitrification and the modeled mainstream deammonification plant.

The construction cost (€) for the individual treatment stages of conventional activated sludge plants with upstream denitrification and mainstream deammonification is shown in Figure 7. The total investment for the new construction of MWWTP with mainstream deammonification is 1.98 Mio. € higher than the conventional plant with upstream denitrification corresponding to approximately 20% higher investment. This is mainly due to the additional stages such as pre-treatment of C removal before mainstream deammonification and downstream treatment of NO_3_^−^ and residual COD removal using the biological activated filter after the secondary sedimentation.

The conventional activated sludge plant with a capacity of 30,000 P.E. in the current study demands 1,050 MWh of electricity for the plant’s operation. Assuming the saving potential of 8.2 kWh//(P.E.∙a) through mainstream deammonification (in the case of 80% efficiency at the pre-treatment C removal stage) according to Kaleß and Pinnekamp (2017) from section 3.2.1, it would yield 246 MWh/a of electricity surplus for an MWWTP with a capacity for 30,000 P.E. These results depict that the concept of mainstream deammonification will be cost-effective in long-term though having 20% higher investment costs.

In addition, the demand for advanced phosphorus elimination in the conventional MWWTPs is to be expected in the future. It has to be evaluated subsequently to what extent a separate phosphorous elimination stage will be required for a conventional wastewater treatment plant. In that case, both concepts of activated sludge and mainstream deammonification are equally comparable within the scope of the accuracy of a cost statement concerning the capital expenditure.

##### 3.2.2.2. Retrofitting the existing MWWTPs for mainstream deammonification

The deammonification process can be operated similarly to the conventional activated sludge process, in which the biomass is suspended as well as granulated. Since the planctomycetes often conglomerate in granular form, it is possible to separate the granules from the sludge by gravity. However, a comparison of the separation technique and efficiency for biomass accumulation is not part of the current study. An estimation of the required reactor volume based on the VNRR in the order of 50 gN/(m^3^∙d) for mainstream deammonification (according to section 2.3) would result in a similar reactor volume as the activated sludge process. Hence, the existing activated sludge reactor basins can be reused when the defined VNRR of 50 gN/(m^3^∙d) is reached by the deammonifying sludge.

Agitators and aeration elements are also essential for the operation of deammonification. Depending on the process of conventional aeration either continuous or interval, it may be necessary to move existing agitators or aeration elements when converting to deammonification. In principle, it should be possible to convert the hydraulics at the plants with the intermittent operation of aeration or with downstream denitrification, in such a way that, partial nitritation can be operated in the existing aerated basins and anammox reaction in the non-aerated zones. However, checking the suitability of existing aeration elements and circulation units is a case-by-case consideration and cannot be generalized. Since the deammonification process requires less oxygen, the aeration units (e.g., blowers or turbo-compressors) can still be used, but are likely to be oversized. Depending on how the blower staging at an MWWTP is set up, smaller units need to be procured. Simultaneously, it is likely to be sufficient to use fewer blowers compared to conventional nitrification/denitrification. It would be a considerable option, especially for larger MWWTPs with several central aeration aggregates and an air supply *via* manifolds.

The existing measurement technology for the conventional activated sludge process can be used for mainstream deammonification. Oxygen measurements and flow measurements may need to be relocated. However, the control of the process must be redesigned depending on the equipment of the existing plant and supplemented by additional quality measurements for the N parameters.

## 4. Conclusion

The comparison conducted between the conventional activated sludge process and mainstream deammonification in the current study is only to be understood as an orientation. The results of laboratory experiments in the current study show that a VNRR of 50 gN/(m^3^∙d) can be achieved by various deammonifying sludges under mainstream temperatures <20°C and pH conditions (6–9). This seems to be also possible in an operationally reliable manner irrespective of seasonal conditions in temperate zones of the earth like Germany. However, a stable VNRR cannot yet be guaranteed as the investigations showed the short-term performance of mainstream deammonification. Therefore, the upcoming studies should focus on the long-term and continuous operation of deammonification reactors, and simultaneous investigations on biological activity of various large-scale deammonifying sludges. The achieved VNRRs by the studied deammonifying sludges in this work meet both the requirements for the reactor volume size and the N elimination capacity in the mainstream of a standard MWWTP of size class of 4 in Germany.

If an MWWTP needs to be operated by consuming less energy, yet yielding a relevant N removal performance in the mainstream, the new construction of a mainstream deammonification plant would be cost-effective in the long term, despite having 20% higher investment costs than the conventional plant.

Based on the results of this study, immediate large-scale implementation of a mainstream deammonification cannot yet be recommended. However, the energy potential of this technology is large and the laboratory results are promising in terms of N removal performance. A scale-up to a semi-technical level should be examined by converting parts of a multi-line activated sludge plant into deammonification.

## Data availability statement

The original contributions presented in the study are included in the article/Supplementary material, further inquiries can be directed to the corresponding author.

## Author contributions

DC: conceptualization and writing–original draft preparation. DC and MG: methodology, formal analysis, and investigation. DC, KG, DM, and MG: writing–review and editing. MG: supervision. All authors have read and agreed to the published version of the manuscript.

## Funding

This study was part of the research project DEA-HS (Einsatz der Deammonifikation zur Stickstoffelimination im Hauptstrom kommunaler Kläranlagen) and it was funded by the Ministry for the Environment, Agriculture, Nature Conservation and Consumer Protection of the State of North Rhine-Westphalia (grant number 17–04.02.01–7/2017). The Open Access publication is sponsored by the equal opportunities office of the FH Aachen.

## Conflict of interest

The authors declare that the research was conducted in the absence of any commercial or financial relationships that could be construed as a potential conflict of interest.

## Publisher’s note

All claims expressed in this article are solely those of the authors and do not necessarily represent those of their affiliated organizations, or those of the publisher, the editors and the reviewers. Any product that may be evaluated in this article, or claim that may be made by its manufacturer, is not guaranteed or endorsed by the publisher.
