# Supplementary material for: Concept development of a mainstream deammonification and comparison with conventional process in terms of energy, performance and economical construction perspectives
Source: Front Microbiol. 2023 Apr 11;14:1155235. doi: 10.3389/fmicb.2023.1155235 (PMC10126410; doi:10.3389/fmicb.2023.1155235)
Supplement: Supplementary file 2 [file Table_1.DOCX]

## Design of the conventional wastewater treatment plant using Design2treat

Table 1: Inflow in the municipal treatment plant

| Inflow in the municipal wastewater treatment plant | Parameter | Value | Unit |
| --- | --- | --- | --- |
|  | Inhabitants | 30,000 | [E] |
|  | Factor | 100 | [%] |
|  | Bypass for pretreatment | 0 | [%] |
|  | Specific wastewater generation (w_s) | 0.125 | [m^3^/(E∙d)] |
|  | External water (w_f) | 938 | [m^3^/d] |
|  | Peak discharge factor for wastewater (x_s) | 12 | [h/d] |
|  | Peak discharge factor for external water (x_f)~~ | 24 | [h/d] |
|  | rain (Q_s) / dry weather for wastewater (Q_s)~~ | 2 | [-] |
|  | rain (Q_f) / dry weather for external water (Q_f)~~ | 1 | [-] |
|  | Dry weather inflow (Q_T) | 352 | [m³/h] |
|  | Mixed water inflow (Q_M) | 664 | [m³/h] |
|  | External water inflow (Q_f) | 39 | [m³/h] |
|  | Daily inflow (Q_d) | 4,688 | [m³/d] |
|  | Q_M / Q_T | 1.89 | [-] |
|  | Q_f / Q_s | 0.13 | [-] |
| Concentrations | BSB_5_ | 0.384 | [kg/m^3^] |
|  | TS0 | 0.448 | [kg/m^3^] |
|  | NH_4_-N | 0.045 | [kg/m^3^] |
|  | N org. | 0.026 | [kg/m^3^] |
|  | KN | 0.07 | [kg/m^3^] |
|  | NO_3_-N | 0 | [kg/m^3^] |
|  | P_ges_ | 0.01 | [kg/m^3^] |
|  | C_CSB_Z | 0 | [kg/m^3^] |
|  | X_CSB_inert_Z | 0 | [kg/m^3^] |
|  | S_CSB_Z | 0 | [kg/m^3^] |
|  | X_CSB_Z | 0 | [kg/m^3^] |
|  | X_inorg_TS_Z | 0 | [kg/m^3^] |
|  | S_CSB_inert_Z | 0 | [kg/m^3^] |
| Loads | BSB_5_ | 1,800 | [kg/d] |
|  | TS0 | 2,100 | [kg/d] |
|  | NH_4_-N | 210 | [kg/d] |
|  | N org. | 120 | [kg/d] |
|  | KN | 330 | [kg/d] |
|  | NO_3_-N | 0 | [kg/d] |
|  | P_ges_ | 45 | [kg/d] |
|  | C_CSB_Z | 0 | [kg/d] |
|  | X_CSB_inert_Z | 0 | [kg/d] |
|  | S_CSB_Z | 0 | [kg/d] |
|  | X_CSB_Z | 0 | [kg/d] |
|  | X_inorg_TS_Z | 0 | [kg/d] |
|  | S_CSB_inert_Z | 0 | [kg/d] |
| Resident-specific values | BSB_5_ | 0.06 | [kg/(E∙d)] |
|  | TS0*** | 0.07 | [kg/(E∙d)] |
|  | NH_4_-N*** | 0.007 | [kg/(E∙d)] |
|  | N org.*** | 0.004 | [kg/(E∙d)] |
|  | KN | 0.011 | [kg/(E∙d)] |
|  | NO_3_-N*** | 0 | [kg/(E∙d)] |
|  | P_ges_.*** | 0.0015 | [kg/(E∙d)] |
|  | C_CSB_Z | 0 | [kg/(E∙d)] |
|  | X_CSB_inert_Z | 0 | [kg/(E∙d)] |
|  | S_CSB_Z | 0 | [kg/(E∙d)] |
|  | X_CSB_Z | 0 | [kg/(E∙d)] |
|  | X_inorg_TS_Z | 0 | [kg/(E∙d)] |
|  | S_CSB_inert_Z | 0 | [kg/(E∙d)] |
| Backloads | Share of KN in ÜS (rX) | 0.5 | [-] |
|  | KN Chargeback in rX | 25.2 | [kg/d] |
|  | Fcktor | 100 | [%] |
|  | Bypass for pretreatment | 0 | [%] |
|  | BSB_5_ | 0 | [kg/d] |
|  | TS0 | 0 | [kg/d] |
|  | NH_4_-N | -31 | [kg/d] |
|  | N org. | 0 | [kg/d] |
|  | KN | -31 | [kg/d] |
|  | NO_3_-N | 0 | [kg/d] |
|  | P_ges_ | 0 | [kg/d] |
|  | C_CSB_Z | 0 | [kg/d] |
|  | X_CSB_inert_Z | 0 | [kg/d] |
|  | S_CSB_Z | 0 | [kg/d] |
|  | X_CSB_Z | 0 | [kg/d] |
|  | X_inorg_TS_Z | 0 | [kg/d] |
|  | S_CSB_inert_AN | 0 | [kg/d] |

Table 2: Pretreatment stage in the municipal wastewater treatment plant

| Pretreatment stage | Parameter | Value | Unit |
| --- | --- | --- | --- |
|  | Type of pretreatment | Primary sedimentation |  |
|  | Volume of primary sedimentation | 352 | [m³] |
|  | Mean flow time at Q_T*** | 1 | [h] |
| Inflow loads at the pretreatment stage | Q_d | 4,688 | [m³/d] |
|  | Q_T | 352 | [m³/h] |
|  | Q_M | 664 | [m³/h] |
|  | BSB_5_ | 1,800 | [kg/d] |
|  | TS0 | 2,100 | [kg/d] |
|  | NH_4_-N | 210 | [kg/d] |
|  | N org. | 120 | [kg/d] |
|  | KN | 330 | [kg/d] |
|  | NO_3_-N | 0 | [kg/d] |
|  | P_ges_ | 45 | [kg/d] |
|  | C_CSB_Z | 0 | [kg/d] |
|  | X_CSB_inert_Z | 0 | [kg/d] |
|  | S_CSB_Z | 0 | [kg/d] |
|  | X_CSB_Z | 0 | [kg/d] |
|  | X_inorg_TS_Z | 0 | [kg/d] |
|  | S_CSB_inert_AN | 0 | [kg/d] |
| Backloads at the pretreatment stage | BSB_5_ | 0 | [kg/d] |
|  | TS0 | 0 | [kg/d] |
|  | NH_4_-N | -31 | [kg/d] |
|  | N org. | 0 | [kg/d] |
|  | KN | -31 | [kg/d] |
|  | NO_3_-N | 0 | [kg/d] |
|  | P_ges_ | 0 | [kg/d] |
|  | C_CSB_Z | 0 | [kg/d] |
|  | X_CSB_inert_Z | 0 | [kg/d] |
|  | S_CSB_Z | 0 | [kg/d] |
|  | X_CSB_Z | 0 | [kg/d] |
|  | X_inorg_TS_Z | 0 | [kg/d] |
|  | S_CSB_inert_AN | 0 | [kg/d] |
| Total inflow of the pretreatment stage | Q_d | 4,688 | [m³/d] |
|  | Q_T | 352 | [m³/h] |
|  | Q_M | 664 | [m³/h] |
|  | BSB_5_ | 1,800 | [kg/d] |
|  | TS0 | 2,100 | [kg/d] |
|  | NH_4_-N | 179 | [kg/d] |
|  | N org. | 120 | [kg/d] |
|  | KN | 299 | [kg/d] |
|  | NO_3_-N | 0 | [kg/d] |
|  | P_ges_ | 45 | [kg/d] |
|  | C_CSB_Z | 0 | [kg/d] |
|  | X_CSB_inert_Z | 0 | [kg/d] |
|  | S_CSB_Z | 0 | [kg/d] |
|  | X_CSB_Z | 0 | [kg/d] |
|  | X_inorg_TS_Z | 0 | [kg/d] |
|  | S_CSB_inert_AN | 0 | [kg/d] |
| Efficiency of the pretreatment stage | BSB_5_ | 24.75 | [%] |
|  | TS0 | 50 | [%] |
|  | NH_4_-N | 0 | [%] |
|  | N org. | 24.75 | [%] |
|  | KN | 0 | [%] |
|  | NO_3_-N | 0 | [%] |
|  | P_ges_ | 14.85 | [%] |
|  | C_CSB_Z | 0 | [%] |
|  | X_CSB_inert_Z | 50 | [%] |
|  | S_CSB_Z | 0 | [%] |
|  | X_CSB_Z | 50 | [%] |
|  | X_inorg_TS_Z | 50 | [%] |
|  | S_CSB_inert_Z | 0 | [%] |
| Outflow at the pretreatment stage | Q_d | 4,688 | [m³/d] |
|  | Q_T | 352 | [m³/h] |
|  | Q_M | 664 | [m³/h] |
|  | BSB_5_ | 1,355 | [kg/d] |
|  | TS0 | 1,050 | [kg/d] |
|  | NH_4_-N | 179 | [kg/d] |
|  | N org. | 90 | [kg/d] |
|  | KN | 269 | [kg/d] |
|  | NO_3_-N | 0 | [kg/d] |
|  | P_ges_ | 38 | [kg/d] |
|  | C_CSB_Z | 0 | [kg/d] |
|  | X_CSB_inert_Z | 0 | [kg/d] |
|  | S_CSB_Z | 0 | [kg/d] |
|  | X_CSB_Z | 0 | [kg/d] |
|  | X_anorg_TS_Z | 0 | [kg/d] |
|  | S_CSB_inert_AN | 0 | [kg/d] |

*Bypasses around the pretreatment stage=Factors and bypasses are included in these loads.

*Inflow #1 bypass around the pretreatment stage; All inflows and loads = zero.

*Backloads bypass around the pretreatment stage; All inflows and loads = zero.

*Sum of bypasses around pretreatment stage; All inflows and loads = zero.

*No external carbon source used

*Loads BB_inflow with no backloads through rX.

Table 3: Lodas at aeration basins

|  | Parameter | Value | Unit |
| --- | --- | --- | --- |
| Inflow loads of aerationbasins with no backloads through rX. (BB_inflow) | Q_d | 4,688 | [m³/d] |
|  | Q_T | 352 | [m³/h] |
|  | Q_M | 664 | [m³/h] |
|  | BSB_5_ | 1,355 | [kg/d] |
|  | TS0 | 1,050 | [kg/d] |
|  | NH_4_-N | 179 | [kg/d] |
|  | N org. | 90 | [kg/d] |
|  | KN | 269 | [kg/d] |
|  | NO_3_-N | 0 | [kg/d] |
|  | P_ges_ | 38 | [kg/d] |
|  | C_CSB_Z | 0 | [kg/d] |
|  | X_CSB_inert_Z | 0 | [kg/d] |
|  | S_CSB_Z | 0 | [kg/d] |
|  | X_CSB_Z | 0 | [kg/d] |
|  | X_inorg_TS_Z | 0 | [kg/d] |
|  | S_CSB_inert_AN | 0 | [kg/d] |
| Concentrations of aeration-basins with no backloads through rX | Q_d | 4,687.50 | [m³/d] |
|  | Q_T | 351.6 | [m³/h] |
|  | Q_M | 664.1 | [m³/h] |
|  | BSB_5_ | 0.289 | [kg/m^3^] |
|  | TS0 | 0.224 | [kg/m^3^] |
|  | NH_4_-N | 0.0382 | [kg/m^3^] |
|  | N org. | 0.0193 | [kg/m^3^] |
|  | KN | 0.0575 | [kg/m^3^] |
|  | NO_3_-N | 0 | [kg/m^3^] |
|  | P_ges_ | 0.00.2 | [kg/m^3^] |
|  | C_CSB_Z | 0 | [kg/m^3^] |
|  | X_CSB_inert_Z | 0 | [kg/m^3^] |
|  | S_CSB_Z | 0 | [kg/m^3^] |
|  | X_CSB_Z | 0 | [kg/m^3^] |
|  | X_anorg_TS_Z | 0 | [kg/m^3^] |
|  | S_CSB_inert_AN | 0 | [kg/m^3^] |
| Outflow values | Monitoring values |  | [kg/m^3^] |
|  | Monitoring values N inorg. | 0.018 | [kg/m^3^] |
|  | Monitoring values NH_4_-N | 0.01 | [kg/m^3^] |
|  | Monitoring values P tot. | 0.001 | [kg/m^3^] |
|  | Design values calculated according to recommendations of LANUV NRW |  | [kg/m^3^] |
|  | Design value N org. on average | 0.002 | [kg/m^3^] |
|  | Rated value NH_4_-N in the peak | 0.01 | [kg/m^3^] |
|  | Rated value NH_4_-N on average | 0.002 | [kg/m^3^] |
|  | Rated value NO_3_-N on average | 0.01 | [kg/m^3^] |

Table 4: Core numbers of aeration basins in the wastewater treatment plant

| Aeration basins | Parameter | Value | Unit |
| --- | --- | --- | --- |
|  | Design approach | University group approach (HSG) |  |
|  | Type of the plant | Upstream denitrification |  |
|  | BB Volume without BioP = V_tot. | 5,189 | [m³] |
|  | Volume of nitrification (V_ni) | 3,285 | [m³] |
|  | Volume of denitrification  (V_deni) | 1,905 | [m³] |
|  | V_deni / V_tot. | 0.367 | [-] |
|  | V_BB-Volumen per inhabitant | 0.173 | [m³]/E] |
|  | V_ni per inhabitant | 0.1095 | [m³]/E] |
|  | V_deni per inhabitant | 0.0635 | [m³]/E] |
|  | Design temperature | 12 | [°C] |
|  | Aerobic sludge age | 8.77 | [d] |
|  | Total sludge age | 13.86 | [d] |
|  | Outlet total solids concentratrion of aeration basins | 3.39 | [kg/m³] |
|  | Mean total solids concentratrion in the aeration basins | 3.39 | [kg/m³] |
|  | Mean biological dry solids concentration | 2.98 | [kg/m³] |
|  | Sludge load BTS (BSB) | 0.077 | [kg BSB /(kg TS∙d)] |
|  | Sludge load BTS (CSB) | 0 | [kg CSB /(kg TS∙d)] |
|  | Rate of denitrification | 0.276 | [kg N /(kg TS∙d)] |
|  | Preset maximum feedback ratio | 6 | [-] |
|  | Calculated feedback ratio | 3.81 | [-] |
|  | Respiration increase factor | 1.27 | [-] |
|  | Acid capacity in the outflow | 55,000 | [mmol/ m³] |
| Sludge fractions | X_H | 0.887 | [kg/m³] |
|  | X_H_BSB | 0.887 | [kg/m³] |
|  | X_H_CSB_ext | 0 | [kg/m³] |
|  | XA | 0.067 | [kg/m³] |
|  | X_I | 2.026 | [kg/m³] |
|  | XF | 0.413 | [kg/m³] |
|  | Excess sludge production | 1,270 | [kg/d] |
|  | Excess sludge fractions |  |  |
|  | X_H | 332 | [kg/d] |
|  | XA | 25 | [kg/d] |
|  | X_I | 758 | [kg/d] |
|  | XF | 155 | [kg/d] |
|  | Specific sludge production of Carbon | n.a. | [kg/d] |
|  | Sludge from Phosphorous elimination | n.a. | [kg/d] |
|  | Denitrification capacity A 131 BSB | n.a. | [-] |
|  | OV C-Value BSB | n.a. | [kg O_2_/kg BSB_5_] |
|  | OV Ni-Value BSB | n.a. | [kg O_2_/kg BSB_5_] |
|  | OV Deni-Value BSB | n.a. | [kg O_2_/kg BSB_5_] |
|  | Denitrification capacity A 131 CSB | n.a. | [-] |
|  | OV C-Value CSB | n.a. | [kg O2/kg CSB] |
|  | OV Ni-Wert CSB | n.a. | [kg O2/kg CSB] |
|  | OV Deni-Wert CSB | n.a. | [kg O2/kg CSB] |
|  | Fluctuating factor HSG | 2 | [-] |
|  | SF Sludge age A 131 | 1.8 | [-] |
|  | Suggestion SF sludge age A 131 | 1.76 | [-] |

Table 5: Phosphorus elimination in the wastewater treatment plant

| Phosphorus elimination | Parameter | Value | Unit |
| --- | --- | --- | --- |
|  | Precipitation with | Eisen(III) - Salt |  |
|  | Spec. precipitant dose | 1.8 | [kg Metall / kg P] |
|  | Spec. precipitant products | 2.5 | [kg TS / kg Metall] |
|  | Spec. Alkaline demand | 60 | [mol / g Metall] |
|  | Spec. FH content | 0 | [kg Metall/kg FM] |
|  | Molar ratio ß~~ | 1.5 | [mol/mol] |
|  | Precipitant dose | 0.0132 | [kg/m³] |
|  | Precipitant demand (nur Metall) | 61.87 | [kg Metall/d] |
|  | Precipitant demand | 0 | [kg FM/d] |
|  | Precipitant products | 154.68 | [kg/d] |

Table 6: Phosphorus and nitrogen balances in the wastewater treatment plant

| Phosphorous balance | Inflow | Value | Unit |
| --- | --- | --- | --- |
|  | P tot. | 38.3 | [kg/d] |
|  | Outflow |  |  |
|  | P tot. reached | 4.7 | [kg/d] |
|  | P incorporated (cell growth) | 10.7 | [kg/d] |
|  | P incorporated (Bio-P) | 0 | [kg/d] |
|  | P precipitated | 22.9 | [kg/d] |
|  | Total | 38.3 | [kg/d] |
| Nitrogen balance | Inflow |  |  |
|  | NH_4_-N | 179 | [kg/d] |
|  | N org. | 90.3 | [kg/d] |
|  | NO_3_-N | 0 | [kg/d] |
|  | KN chargeback from rX | 25.2 | [kg/d] |
|  | Total | 294.5 | [kg/d] |
|  | Outflow |  |  |
|  | NH_4_-N | 9.4 | [kg/d] |
|  | N org. | 9.4 | [kg/d] |
|  | NO_3_-N | 46.9 | [kg/d] |
|  | N_2_ through BSB or CSB | 178.5 | [kg/d] |
|  | N_2_ by ext. carbon source | 0 | [kg/d] |
|  | KN Incorporated | 50.4 | [kg/d] |
|  | Total | 294.5 | [kg/d] |

Table 7: Aerator design Impact factors in the wastewater treatment plant

| Aerator sizing Apply impact factors individually (fC=X;fN=1 \| fC=1;fN=Y) | Parameter | Value | Unit |
| --- | --- | --- | --- |
|  | O_2_ - Konzentration | 0.002 | [kg/m^3^] |
|  | alpha - value | 0.7 | [-] |
|  | Impact factor fC | 1.5 | [-] |
|  | Impact factor fN | 1.2 | [-] |
|  | Air pressure | 101.3 | [kPa] |
|  |  |  |  |
|  | Load case 1 | Ni/Deni bei 10°C |  |
|  | Design temperature  O_2_-entry | 10 | [°C] |
|  | Decisive | Impact factor fC |  |
|  | O_2_- Saturation Conc. | 0.011.28 | [kg/m^3^] |
|  | OVC - Value | 1.19 | [kg O_2_/kg BSB_5_] |
|  | OVN - Value | 0.38 | [kg O_2_/kg BSB_5_] |
|  | OVDN - Value | n.a. | [kg O_2_/kg BSB_5_] |
|  | OB - Value | 2.64 | [kg O_2_/kg BSB_5_] |
|  | Hourly peak O_2_ demand | 213.24 | [kg/h] |
|  | Daily demand O_2_ | 3,712.47 | [kg/d] |
|  |  |  |  |
|  | Load case 2 | Ni/Deni at 20°C |  |
|  | Design temperature O_2_-entry | 20 | [°C] |
|  | Decisive | Impact factor fC |  |
|  | O_2_- Saturation Conc | 0.00908 | [kg/m^3^] |
|  | OVC - Value | 1.33 | [kg O_2_/kg BSB_5_] |
|  | OVN - Value | 0.38 | [kg O_2_/kg BSB_5_] |
|  | OVDN - Value | n.a. | [kg O_2_/kg BSB_5_] |
|  | OB - Value | 3.06 | [kg O_2_/kg BSB_5_] |
|  | Hourly peak O_2_ demand | 246.61 | [kg/h] |
|  | Daily demand O_2_ | 4,262.92 | [kg/d] |
|  |  |  |  |
|  | Lastfall 3 | Nitri/Deni bei Bemessungstemperatur | |
|  | Design temperature O_2_-entry | 12 | [°C] |
|  | Decisive | Stoßfaktor fC |  |
|  | O_2_- Saturation Conc | 0.01077 | [kg/m^3^] |
|  | OVC - Value | 1.23 | [kg O_2_/kg BSB_5_] |
|  | OVN - Value | 0.38 | [kg O_2_/kg BSB_5_] |
|  | OVDN - Value | n.a. | [kg O_2_/kg BSB_5_] |
|  | OB - Value | 2.73 | [kg O_2_/kg BSB_5_] |
|  | Hourly peak O_2_ demand | 220.02 | [kg/h] |
|  | Daily demand O_2_ | 3,824.12 | [kg/d] |

Table 8: Calculation of the secondary settling tanks in the wastewater treatment plant

| NKB-Group1 | Calculation of secondary sedimentation tanks | Dimensioning according to ATV-DVWK-A 131 (5/2000) | |
| --- | --- | --- | --- |
|  | NKB - Type | Horizontal flow basins | |
|  | Parameter | Value | Unit |
|  | Default surface | n.a. | [m²] |
|  | Surface | 541 | [m²] |
|  | Share of feed | 0 | [%] |
|  | NKB – Feeding at Q_M | 664 | [m³/h] |
|  | Surface feeding q_A | 1.23 | [m/h] |
|  | Sludge volume index ISV | 120 | [l/kg] |
|  | Sludge volume feed q_SV | 500 | [l/(m²*h)] |
|  | Thickening time te | 2.5 | [h] |
|  | RV at Q_M | 0.75 | [-] |
|  | Average RV bei Q_M | 0.75 | [-] |
|  | Ratio TS_RS / TS_BS | 0.7 | [-] |
|  | TS in RS | 7.92 | [kg/m³] |
|  | TS in the effluent of the aeration basins | 3.39 | [kg/m³] |
|  | Delta TS [TS_BB - TS_AB] | 0 | [kg/m³] |
|  | TS in the influent of the secondary sedimentation tanks | 3.39 | [kg/m³] |
|  | Default 2/3 depth | n.a. | [m] |
|  | 2/3 depth | 4.71 | [m] |
|  | Clear water zone h1 | 0.5 | [m] |
|  | Separation zone h2 | 1.81 | [m] |
|  | Storage zone h3 | 0.79 | [m] |
|  | Thickening and clearing zone h4 | 1.61 | [m] |

Table 9: Kinetic parameters in the wastewater treatment plant

| Kinetic Parameter | Parameter | Value | Unit |
| --- | --- | --- | --- |
|  | µ max,A~~ | 0.52 | [1/d] |
|  | KN~~ | 0.0007 | [kg N/m^3^] |
|  | b_A~~ | 0.05 | [1/d] |
|  | b_H~~ | 0.17 | [1/d] |
|  | f_T,A~~ | 1.103 | [-] |
|  | f_T,bA~~ | 1.09 | [-] |
|  | f_T,bH~~ | 1.073 | [-] |
|  | Y_A~~ | 0.15 | [kgTS/kgN] |
|  | Y_H~~ | 0.75 | [kgTS/kgBSB_5_] |
|  | f_D~~ | 0.75 | [-] |
|  | f_P~~ | 0.6 | [-] |
|  | i_B~~ | 0.12 | [-] |
|  | i_I~~ | 0.01 | [-] |
|  | i_P~~ | 0.03 | [-] |
|  | f_I~~ | 0.2 | [-] |
|  | f_OV,S~~ | 0.56 | [-] |
|  | f_OV,e~~ | 0.2 | [kgO_2_/(kgTS∙d)] |
|  | f´~~ | 1.25 | [-] |
|  | Eta BSB5~~ | 0.95 | [-] |
|  | N incl./BSB5 (A 131)~~ | 0.043 | [-] |
|  | N incl./CSB (A 131)~~ | 0.021 | [-] |
|  | P incl./BSB5 (A 131)~~ | 0.01 | [-] |
|  | P incl./CSB (A 131)~~ | 0.005 | [-] |

*Factors and bypasses are not included in these values.

***entered by the user

~~corresponds to the default value

n.a. not applicable
